# Improvements in Hemodynamics and Right Heart Remodeling Following Balloon Pulmonary Angioplasty Treatment in Patients With Chronic Thromboembolic Pulmonary Hypertension: A Retrospective Study

**DOI:** 10.1002/hsr2.70384

**Published:** 2025-01-22

**Authors:** Ahmad Furqan Anjum, Moeeza Fatima, Muhammad Burhan Anjum

**Affiliations:** ^1^ Shaikh Khalifa Bin Zayed Al‐Nahyan Medical College Shaikh Zayed Postgraduate Medical Institute (SZPGMI) Sahiwal Punjab Pakistan; ^2^ Shaikh Khalifa Bin Zayed Al‐Nahyan Medical College Shaikh Zayed Postgraduate Medical Institute (SZPGMI) Vehari Punjab Pakistan; ^3^ Akhtar Saeed Medical and Dental College Lahore Pakistan

Dear Editor,

This recent study, “Improvements in Right Heart Remodeling and Hemodynamics after Balloon Pulmonary Angioplasty (BPA) Treatment in Patients with Chronic Thromboembolic Pulmonary Hypertension (CTEPH)–A Retrospective Study” by Shen et al. [1], is worth reading because of the insight it provides into improvements to the patients with CTEPH. The findings here suggest that BPA might be a useful means to improve clinical and hemodynamic outcomes in a difficult subset of patients. This is especially important as to date, no therapeutic options are available for patients not eligible for pulmonary endarterectomy (PEA). The improvements they reported appear promising, but a few things about their study need to be considered more carefully before lending credence to their claims.

First, in this study, conventional echocardiographic parameters like right atrial area (RAA) and right ventricular internal diameter (RVID), along with the tricuspid annular plane systolic excursion (TAPSE), are used. However, these metrics have shortcomings that limit the ability to fully capture the picture of RV remodeling and mechanics, especially because they need to be more in line with more subtle underlying functional or electromechanical changes. Kanar et al. [2] undertook a more detailed study of RV function using speckle tracking echocardiography (STE) to quantify electromechanical delay and strain in relation to the regional and temporal mechanics of RV. Second, the study also requires a control group. Having a control group contextualizes the findings and makes it easier to evaluate the observed changes as BPA specific rather than a part of natural variability. A study by Kanar et al. [2] includes the comparison of a group of healthy subjects so that baseline differences can be clearly established and the specific therapeutic effects of BPA can be laid. Third, the study restricts hemodynamic assessments to resting conditions and assumes that exercise‐induced limitations in pulmonary and cardiac function will be manifested. Therefore, it does not measure exercise capacity, and the associated hemodynamic changes during periods of exercise, that are the key features in understanding functional outcomes and residual disease burden. No doubt, the study reports significant improvements in resting hemodynamics but does not address whether residual pulmonary hypertension (PH) exists during physical exertion. Thus, without exercise‐specific data, The study cannot comment on the persistence of exercise‐induced PH or its clinical implications. For example, a study by Wiedenroth et al. [3] incorporates exercise right heart catheterization (RHC) to evaluate pulmonary pressures, vascular resistance, and cardiac output during physical exertion. This approach identifies residual PH during exercise, even in patients without PH at rest, providing a dynamic assessment of BPA efficacy. It also demonstrates that BPA reduces resting mPAP but does not fully normalize exercise hemodynamics, highlighting residual disease and the need for ongoing medical therapy. Fourth, the study does not address how baseline characteristics, such as pulmonary comorbidities, influence BPA outcomes. It treats the patient cohort as homogeneous. The study could have benefited from subgroup analyses to identify differential responses based on patient‐specific factors (e.g., comorbidities, functional capacity, or baseline hemodynamic profiles). For example, a study by Wang et al. [4] specifically examines the impact of baseline pulmonary comorbidities, providing insights into heterogeneous responses to BPA. It finds out that while BPA improves hemodynamics and right heart function across all patients, those without pulmonary comorbidity have better improvements in exercise capacity and respiratory function. Fifth, the study reports remodeling outcomes at 3 and 6 months but does not assess immediate postprocedure changes. By excluding early treatment assessments, the study fails to assess the temporal dynamics of remodeling induced by BPA and cannot distinguish between rapid and delayed treatment effects. In doing so, important insights into the efficacy of BPA are overlooked in the immediate postprocedure period, which can inform both monitoring and subsequent adjustments. A study by Ding et al. [5] found that structural inverse remodeling preceded functional remodeling and noticed that, within 24 h after BPA, no RV systolic function parameters (e.g., TAPSE and RVFAC) showed any significant improvement. Additionally, it highlights rapid post‐BPA changes in RV remodeling and minimal overall recovery in RV systolic function within 24 h.

Given these gaps, we suggest that future research take a broader approach to evaluating BPA outcomes. This strain will improve the sensitivity of the assessments of RV remodelling and function by integrating advanced imaging modalities, including speckle‐tracking echocardiography (STE). Adding a control group, with either just healthy subjects or subjects with other alternative treatments, will enable a more robust comparison and understanding of the BPA‐specific benefits. Furthermore, incorporating exercise right heart catheterization (RHC) and functional capacity metrics, such as the 6‐min walk distance (6MWD) test, would provide critical insights into the persistence of exercise‐induced PH and its clinical implications. Subgroup analyses, particularly those accounting for baseline comorbidities or varying degrees of PH severity, could better elucidate differential responses to BPA and inform patient selection criteria. Lastly, adding early post‐procedure assessments alongside long‐term follow‐ups would capture the timeline of hemodynamic and structural changes, enabling optimized post‐BPA management.

In conclusion, Shen et al. have made an important contribution to the field by demonstrating the potential benefits of BPA in CTEPH patients. However, by addressing the gaps above, future studies can refine our understanding of BPA's efficacy, ensuring its maximum benefit to diverse patient populations. These enhancements would not only strengthen the evidence base for BPA but also support more tailored and effective treatment strategies for CTEPH patients, ultimately improving their quality of life and clinical outcomes.

## Author Contributions

**Ahmad Furqan Anjum:** conceptualization, validation, writing–original draft, writing–review and editing, data curation, supervision, project administration. **Moeeza Fatima:** writing–review and editing. **Muhammad Burhan Anjum:** writing–review and editing.

## Conflicts of Interest

The authors declare no conflicts of interest.

## Transparency Statement

The lead author Ahmad Furqan Anjum affirms that this manuscript is an honest, accurate, and transparent account of the study being reported; that no important aspects of the study have been omitted; and that any discrepancies from the study as planned (and, if relevant, registered) have been explained.

## Data Availability

The authors have nothing to report.
